# Construction and evaluation of *Brassica rapa* orphan genes overexpression library

**DOI:** 10.3389/fpls.2025.1532449

**Published:** 2025-01-22

**Authors:** Mingliang Jiang, Zongxiang Zhan, Xiaonan Li, Zhongyun Piao

**Affiliations:** ^1^ Molecular Biology of Vegetable Laboratory, College of Horticulture, Shenyang Agricultural University, Shenyang, China; ^2^ School of Agriculture, Jilin Agricultural Science and Technology University, Jilin, China

**Keywords:** *Brassica rapa*, orphan genes, overexpression library, construction, evaluation

## Abstract

Orphan genes (*OGs*) are crucial for species-specific characteristics and stress responses and are restricted to a specific taxon. However, their functions within particular species are poorly understood. Previous research identified *OGs* in *Brassica rapa* (*BrOGs*). In this study, the *BrOGs* overexpression (BrOGsOE) library in *Arabidopsis thaliana* was constructed. Approximately 128 unknown functional *BrOGs* were selected from Chinese cabbage and were overexpressed. The analysis focused on the phenotypes of leaf morphology and flowering time against phenotypic differences between Chinese cabbage and *Arabidopsis*. Interestingly, 72.66% of the transgenic lines showed distinctive phenotypic changes. Chinese cabbage-specific features, including curved, hairy, upward or downward-curving leaves, serrated margins, and multiple leaves, were observed in the BrOGsOE lines. The *BrOGs* overexpression library was associated with numerous variations in flowering time, particularly delayed flowering. This suggested that the delayed flowering time caused by *BrOGs* may be associated with resistance to bolting seem in Chinese cabbage. Furthermore, the results of stress treatment of 24 BrOGsOE lines with no apparent significant phenotypes suggested that a number of *BrOGs* have both general and specific functions against environmental and pathogenic stress. The findings of this study provide a comprehensive overview of the roles of *BrOGs*, emphasizing their significance as a resource for identifying positive genes associated with species-specific characteristics and stress responses and offering a solid foundation for the functional analysis of *BrOGs*.

## 1 Introduction

Orphan genes (*OGs*), known as species-specific genes, are characterized by their lack of detectable resemblance to other proteins and are prevalent across nearly all organisms (Jiang et al., 2022; Moreyra et al., 2023). They arise within a single species or a taxonomically confined gene family formed by expressing unique open reading frames (ORF). They are present throughout evolutionary history (Liu et al., 2023). These *OGs* provide organisms with a reservoir of genetic components that enable rapid responses to altering selection pressures, serving as a disruptive factor in evolution and playing a vital role in adaptation to novel biological niches (Li et al., 2022). These genes have been found and described in several plants, including *A. thaliana* (Lin et al., 2010), *Populus trichocarpa* (Lin et al., 2013), *Citrus sinensis* (Xu et al., 2015), and *B. rapa* (Jiang et al., 2018). The results of these investigations provide references for the comprehensive analysis of *OGs*.

Considering the challenges in examining *OGs* due to their lack of comparability with proteins from other lineages (Kimmel et al., 2023), specific studies on the functions of *OGs* provide key basic references. These genes are frequently associated with responses to stress, particular characteristics of the species, the regulation of specialized genes, and fundamental metabolic processes (Jiang et al., 2022). Several *B. rapa OGs* (*BrOGs*) have been found to be essential for soluble sugar metabolism, with *B. rapa OG 1* (*BrOG1*) apparently regulating this process in a sucrose synthase (SUS)-dependent manner (Jiang et al., 2020). Other *BrOGs*, such as *BOLTING RESISTANCE 1* (*BR1*), have been shown to influence flowering time in *Arabidopsis*, potentially functioning *via* vernalization and photoperiodic pathways (Jiang et al., 2023). A second *OG* found from *B. rapa*, termed *BOLTING RESISTANCE 2* (*BR2*), positively modulates bolting resistance in both *Arabidopsis* and Chinese cabbage, potentially functioning through the vernalization pathway (Zu et al., 2024). Knockouts of the *Zea* genus-specific micropeptide microRPG1 encoded by the *qKDR1 REGULATED PEPTIDE GENE* (*RPG*) locus induces a faster kernel dehydration rate (KDR) in maize (Yu et al., 2024). A bean orphan protein MRE-binding transcription factor 1 (PvMTF-1) is related to a metal-responsive element involved in cadmium resistance in transgenic tobacco (*Nicotiana tabacum*) through activation of tryptophan biosynthesis (Sun et al., 2015). *Physcomitrium patens OG ABA-responsive drought tolerance* (*PpARDT*) imparts drought tolerance in terrestrial plants, possibly by promoting the ABA response, thereby elucidating the functions of *OG* in affecting lineage-specific adaptation, probably through the recruitment of pre-existing pathway components (Dong et al., 2022). The *Arabidopsis OG QQS* has been confirmed as a regulator of carbon and nitrogen partitioning in various species through interactions with Nuclear Factor Y subunit C (NF-YC), as well as influencing both stress responses and pollen germination and viability (Li and Wurtele, 2015; Li et al., 2015; Fakhar et al., 2023; Luo et al., 2024). Recently, CRISPR/Cas9-based editing of *NF-YC4* promoters to increase rice and soybean protein yields has shown that NF-YC4 interacts with QQS, paving the way for improved crop productivity and nutritional value (Wang et al., 2024). *Populus trichocarpa OG BOOSTER* (*BSTR*) has been found to impact photosynthesis, and overexpression of *BSTR* improved biomass gains in poplar and *Arabidopsis* (Feyissa et al., 2024). However, the functions of *BrOGs* are not well identified.

The genomes of *B. rapa* and the closely associated *A. thaliana* have been invaluable resources for studying genomic evolution. *B. rapa* is planted extensively throughout the world due to its highly varied morphological characteristics, which have significant economic and breeding value. These characteristics include the leafy heads of Chinese cabbage, the oversized organs of turnip, and the broad axillary branching of Pak-choi (Song et al., 2014). After the emergence of *Arabidopsis*, the *B. rapa* genome underwent diversification approximately 12.4 to 13.4 million years ago (Yang et al., 2006; Liu et al., 2014; Waminal et al., 2016). The reasons underlying the evolution of varieties with large phenotypic differences within such a relatively short period are not fully understood, and the mechanisms associated with the speciation and morphological diversification of *B. rapa* in response to both natural and artificial selection require further investigation. Various *BrOGs* were identified and characterized in a previous study (Jiang et al., 2018). However, further research is needed into the potential contributions of these newly emerged *BrOGs* to the complex morphological characteristics of *B. rapa*, together with their possible associations with species-specific adaptation and stress responses.

This study constructed and characterized an overexpression library of *BrOGs* in *A. thaliana* to enable the enhancement of *BrOG* functions and the discovery of genes. The phenotypes of the BrOGsOE lines were then examined. As *OGs* are frequently associated with various forms of stress (Jiang et al., 2022), BrOGsOE lines that were not associated with distinctive phenotypic variations were challenged with biotic and abiotic stressors. The findings of this study provide a detailed analysis of the roles of *BrOGs* in species-specific characteristics and responses to stress.

## 2 Materials and methods

### 2.1 Plant materials and growth conditions

The *A. thaliana* ecotype Col-0 (WT) and the transformed lines were allowed to grow in a long-day (LD) environment (16 h light/8 h dark cycles) under cool white fluorescent light at 22°C with 65-70% humidity. To identify *BrOGs* at DNA and expression levels in BrOGsOE plants, seedlings of the WT and BrOGsOE lines from 9 individuals (three biological replicates with three plants per replicate) were obtained for DNA and RNA extraction after two weeks of growth.

### 2.2 Plant vector constructions, transformation, and *Arabidopsis* BrOGsOE lines selection

According to the previous study, procedures for plant vector constructions, transformation, and *Arabidopsis* BrOGsOE lines selection were performed as described previously (Jiang et al., 2020). Homozygous BrOGsOE lines were identified *via* the DsRed protein in seeds using a combination of red fluorescent protein excitation light and filter. The sequences of the forward and reverse primers used for the vector constructs are provided in **Supplementary Table S1**.

### 2.3 Characterization of transgenic lines

For the characterization of BrOGsOE lines, flowering time, rosette radius, silique length, seed number, stem height, leaf shape, and fertility were measured as per the previously established protocols (Jiang et al., 2020). A total of 15 plants of every BrOGsOE line or WT were examined.

### 2.4 Pathogen inoculations and quantification

For pathogen stress treatment, 4-5-week-old WT and BrOGsOE lines were hand-infiltrated with *Pseudomonas syringae* pv. *tomato* DC3000 (*Pst* DC3000) bacterial suspensions (OD_600_ = 0.0002 in 10 mM MgCl_2_), and the bacterial load was quantified at 3 dpi. *Pst* DC3000 was cultured as previously described (Nomura et al., 2012). Images were captured at 3 dpi. Three biological replicates were scored with at least 12 plants per replicate.

### 2.5 Salt- and heat-stress treatment

Seeds of WT and T_2_ homozygous BrOGsOE lines were surface-sterilized with bleach, washed 5 times with sterile water, and seeded on 1/2 MS medium containing 1/2 Murashige and Skoog Basal Medium with Vitamins (PhytoTech, KS, US) and 0.8% (w/v) agar. Control plates were incubated in the dark for 3 days at 4°C and then grown at 22°C under LD conditions. For salt treatment, seeds of the WT and BrOGsOE plants were cultured on 1/2 MS agar medium plates and grown at 4°C for 3 days with either 0 or 150 mM NaCl. Images were captured after 9 days of incubation at 22°C. For heat stress, the plates were kept at 4°C for 3 days, followed by incubation at 22°C for 30 h with a further incubation at 45°C for 2 h, recovered and allowed to grow at 22°C for 6 days before imaging. The survival rates after salt and heat stress were calculated from the number of seedlings with green, expanded cotyledons; three biological replicates were scored with 16 to 18 plants per replicate.

### 2.6 DNA and RNA isolation, cDNA synthesis, PCR, semi-quantitative RT-PCR and qRT-PCR

All DNA and RNA extractions, cDNA synthesis, PCR, RT-PCR, and qRT-PCR were performed as described previously (Jiang et al., 2018). The *AtACTIN2* (*AT3G18780*) gene was used as the housekeeping gene for semi-quantitative RT-PCR to verify the BrOGsOE transgenic lines (Lu et al., 2012). The *AtPP2AA3* (*At1G13320*) gene was used as the internal control for qRT-PCR analysis of *A. thaliana Pathogenesis-related gene 1* (*AtPR1*) after *Pst* DC3000 treatment (Huot et al., 2017). Gene expression was calculated using the 2^-ΔΔCt^ method (Livak and Schmittgen, 2001). The experiment was carried out in three biological sets. The sequences of all the forward and reverse primers are provided in **Supplementary Table S1**.

### 2.7 Statistical analysis

Data were analyzed with SPSS v19.0 software using Student’s *t*-test or one-way ANOVA followed by individual comparisons with Duncan’s multiple range test. GraphPad Prism v8.0.2 and TBtools-II v2.119 (Chen et al., 2023) software were used for illustrations.

## 3 Results

### 3.1 Phenotypic variations between Chinese cabbage and *A. thaliana*

**Figure 1:**
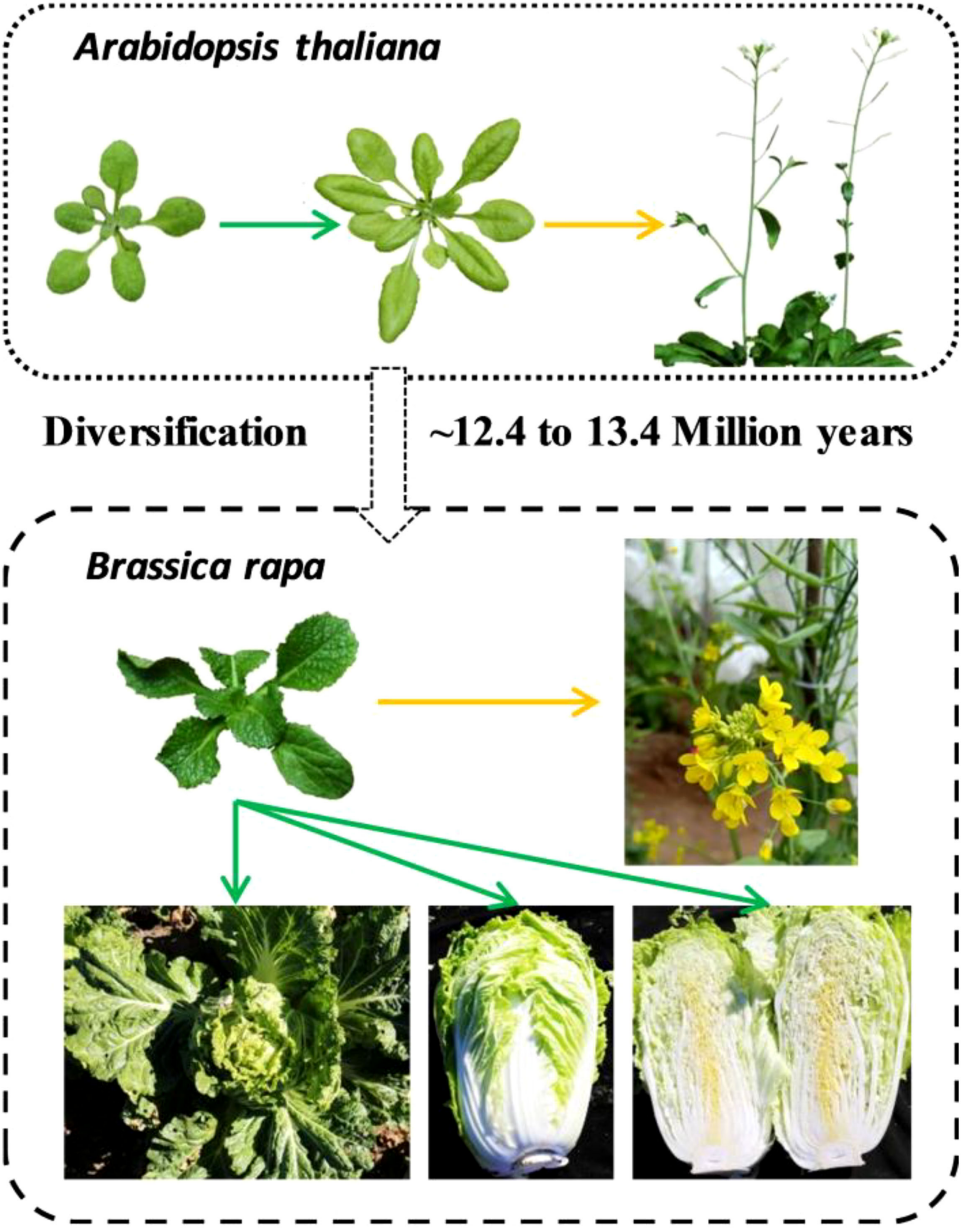
Comparison of evolutionary relationships and phenotypic differences between Chinese cabbage and *A. thaliana*. Chinese cabbage has wavy margins, serrated blades with uneven surfaces, and downward or upward-curved leaves, in contrast to the flat leaves of *A. thaliana*. Chinese cabbage requires several weeks of exposure to cold (vernalization) to induce flowering, whereas *Arabidopsis* displays only a small vernalization response. The green arrow represents vegetative growth, and the yellow arrow represents reproductive growth.

Plants controlled by developmental genetic programming show species-specific features. Despite the close relationship between *A. thaliana* and Chinese cabbage, substantial variation is seen in different phenotypic characteristics, including leaf morphology, leaf size, and flowering phenotype. Heading Chinese cabbage possesses a leafy head characterized by very inwardly curled blades at the shoot apex, and the leafy head consists of multiple heading leaves that typically curve inwards after the rosette stage (Ren et al., 2018). The multiple leaves with serrated margins and downward or upward curves contrast sharply with the relatively fewer and flat leaves of *A. thaliana* (**Figure 1**). In the life cycles of flowering plants, the primary developmental transition is from vegetative to reproductive growth (Domagalska et al., 2007). Chinese cabbage is a late-flowering plant that requires several weeks of exposure to low temperatures (a process known as vernalization) to induce flowering, whereas *Arabidopsis* displays only a small vernalization response (Sheldon et al., 2000; Yuan et al., 2009).

Collectively, the study into the leaf morphology and flowering phenotype of Chinese cabbage holds considerable importance for breeding. However, within a short evolutionary timeframe, the phenotypes of Chinese cabbage and *Arabidopsis*, have diverged markedly, suggesting that *BrOGs*, as newly evolved genes, may play a significant role in phenotypic regulation. Due to the limited data available on *BrOGs* function, bioinformatics analysis is an effective means of acquiring more information. This led to the development of a transgenic library of *BrOGs*.

### 3.2 Construction of the *BrOGs* overexpression library and phenotypic observations

For a better understanding of *BrOGs* functions, a *BrOGs* overexpression library was constructed in *Arabidopsis*. One hundred and twenty-eight unknown functional Chinese cabbage *BrOGs* were successfully transformed into *Arabidopsis* by floral dip transformation with random selection, including 43 *BrOGs* that were successfully transformed in previous studies (Jiang et al., 2020) and have been summarized and analyzed in this article. These 128 *BrOGs* were identified in a previous study (Jiang et al., 2018). The expression of the *BrOGs* was regulated by the Cauliflower Mosaic Virus 35S (CaMV35S) promoter (**Supplementary Figure S1**). The addition of the *DsRed* gene regulated by the CaMV35S promoter enhances the accessibility and efficiency of screening transgenic plants (Zhang et al., 2013). After the floral dip transformation, the T_2_ homozygous seeds derived from various self-pollinated T_1_ transgenic seed lines were identified *via* the *DsRed* gene. To further confirm the correctness of the BrOGsOE plants, 10 BrOGsOE plants and WT were randomly selected for verification at DNA and expression levels. Both genomic and CDS sequences were successfully amplified in the BrOGsOE lines (**Supplementary Figure S2**) and there were no target strips in the WT. Furthermore, target bands were sequenced, confirming the accuracy of the *BrOGs* sequences. The study then, considering the specific phenotypes of Chinese cabbage compared to *A. thaliana* (**Figure 1**), focused on the characteristics of leaf shape and flowering time.

**Figure 2:**
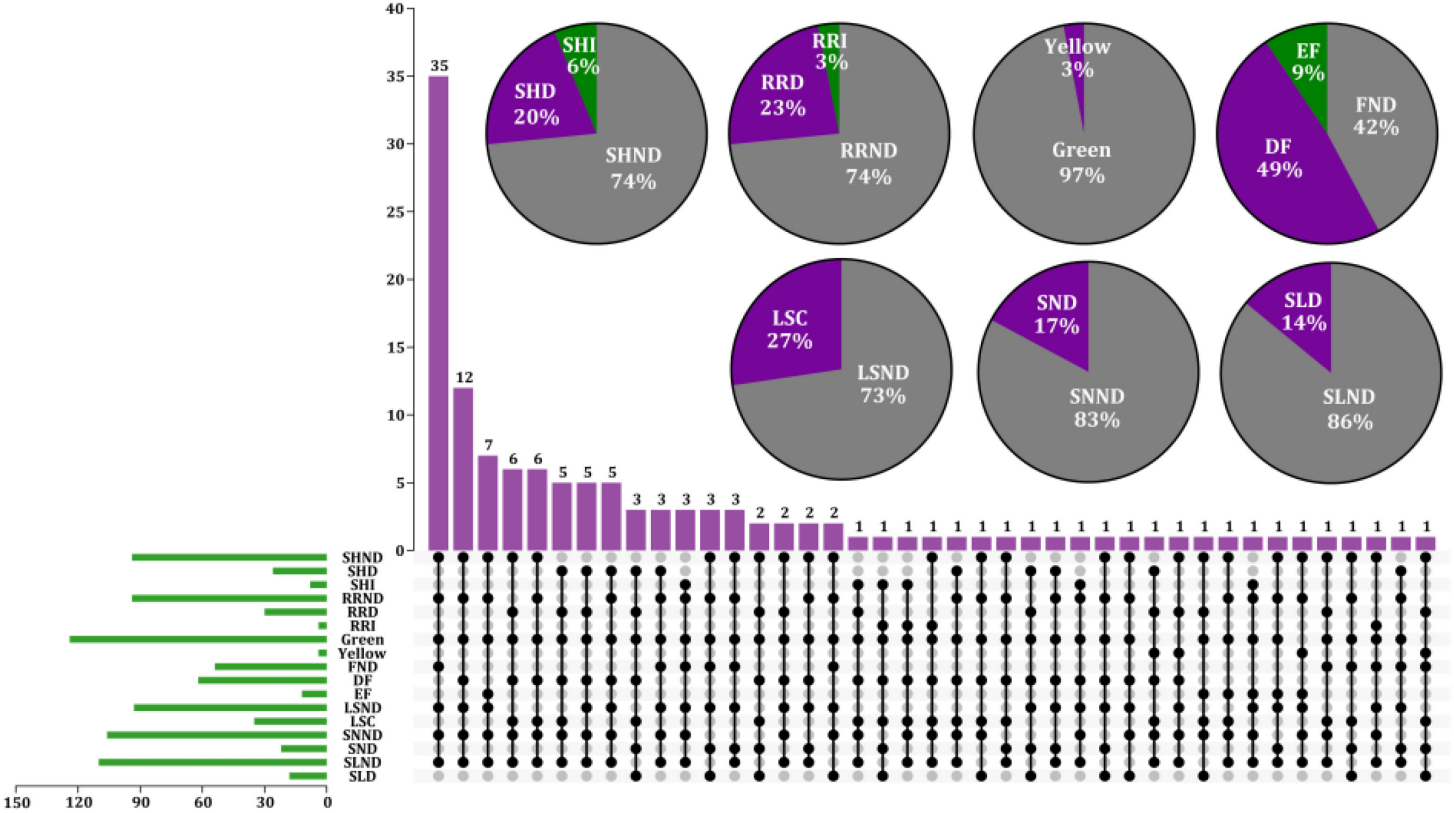
Characterization of different transgenic line types and phenotype analysis of 128 BrOGsOE lines. Different BSGsOE lines showed both unique and shared phenotypes. The purple column represents the summary of the various phenotypes of different transgenic plants. The gray solid circle indicates that the transgenic plant has no corresponding phenotype. The green column in the lower left corner depicts the total number of transgenic plants respective to phenotype. The pie charts in the upper right corner show the percentage of single phenotypes, including stem height, rosette radius, leaf color, flowering time, leaf shape, seed number, and silique length. SHND indicates stem height no difference when compared with WT; SHD and SHI indicate stem height decrease and increase, respectively; RRND indicates rosette radius no difference; RRD and RRI indicate rosette radius decrease and increase, respectively; Leaf color is represented by Green and Yellow; FND indicates no difference in flowering time; DF and EF indicate delayed and early flowering, respectively; LSND indicates leaf shape no difference; LSC indicates leaf shape change; SNND indicates seed number no difference; SND indicates seed number decrease; SLND indicates silique length no difference; SLD indicates silique length decrease.

Phenotypic changes in the BrOGsOE lines were investigated, focusing specifically on leaf morphology, flowering time, and other characteristics. The following traits were specifically analyzed during the vegetative and reproductive stages: flowering duration, stem height, rosette radius, leaf shape and color, silique length, and seed number. Stable homozygous T_2_ transgenic plants were used for observation. A total of 93 BrOGsOE lines with phenotype variations were obtained, accounting for 72.66%, and no phenotypic variation was found in the remaining 35 BrOGsOE lines (27.34%) (**Figure 2**).

**Figure 3:**
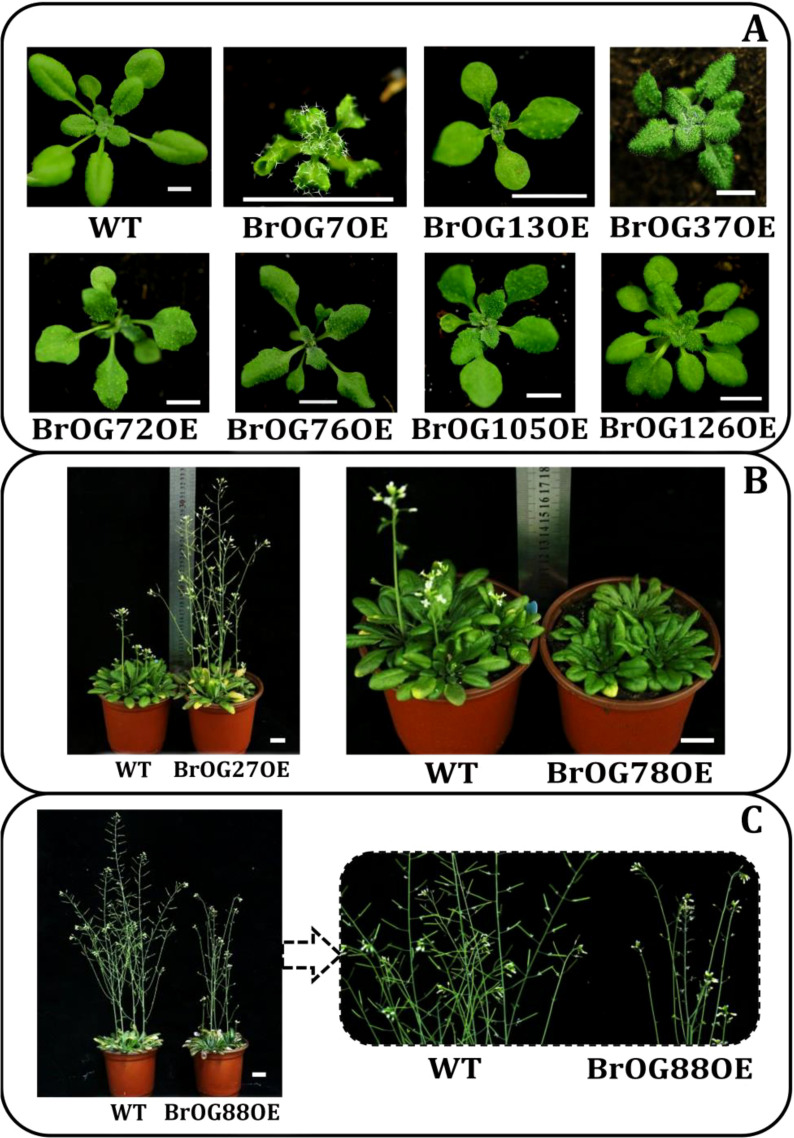
Visible transgenic lines appearing in phenotypes of *BrOGs* over-expression (OE) *Arabidopsis* lines. WT indicates *A. thaliana* Col-0. These phenotypes are represented in the T_2_ generations. Representative individuals are shown at **(A)** 22 days (Bar = 0.5 cm), **(B)** 33 days, and **(C)** 45 days of growth (Bar = 2 cm).

The delayed flowering phenotype was relatively common in the transgenic library, comprising approximately 49% of the samples. The early flowering phenotype was represented by 9% of the samples, and approximately 42% of the transgenic plants exhibited no discernible difference in flowering time (**Figures 2**, **3**). The results of the investigation of representative extremely significant phenotypes of BrOGsOE lines are shown in **Supplementary Table S2**. Notably, 49% of the BrOGsOE lines showed a delayed flowering phenotype, with some lines showing various other phenotypes, including reduced or increased rosette radius, increased or decreased stem height, variations in leaf shape, decreased silique length, fewer seed numbers, and yellow leaf color. For example, BrOG72OE, BrOG76OE, and BrOG105OE all showed delayed flowering. The leaves of these transgenic plants were characterized by more uneven leaf surfaces and greater serration of the leaf edges relative to the WT. The BrOG126OE transgenic plants exhibited the delayed flowering phenotype accompanied by a reduced rosette radius and a significant increase in rosette leaves. The percentage of the delayed flowering type was markedly higher than that of the early flowering type, and delayed flowering was accompanied by additional phenotypic features.

The phenotypes of the BrOG7OE transgenic plants included an upward-curled leaf and an increase in leaf hair (**Figure 3A**). BrOG78OE displayed a delayed flowering phenotype, lower rosette radius, and curled leaves (**Figure 3B**). BrOG88OE also showed a delayed flowering phenotype, together with reduced stem height, a shortened silique length, and fewer seeds per silique (**Figure 3C**). Moreover, 35 *Arabidopsis* lines were identified that showed visible changes in leaf shape, including BrOG13OE and BrOG37OE. This suggests a clear correlation between the leaf shape of these *Arabidopsis* lines and the specific leaf characteristics of Chinese cabbage.

### 3.3 Screening of pathogen stress-response BrOGsOE lines

**Figure 4:**
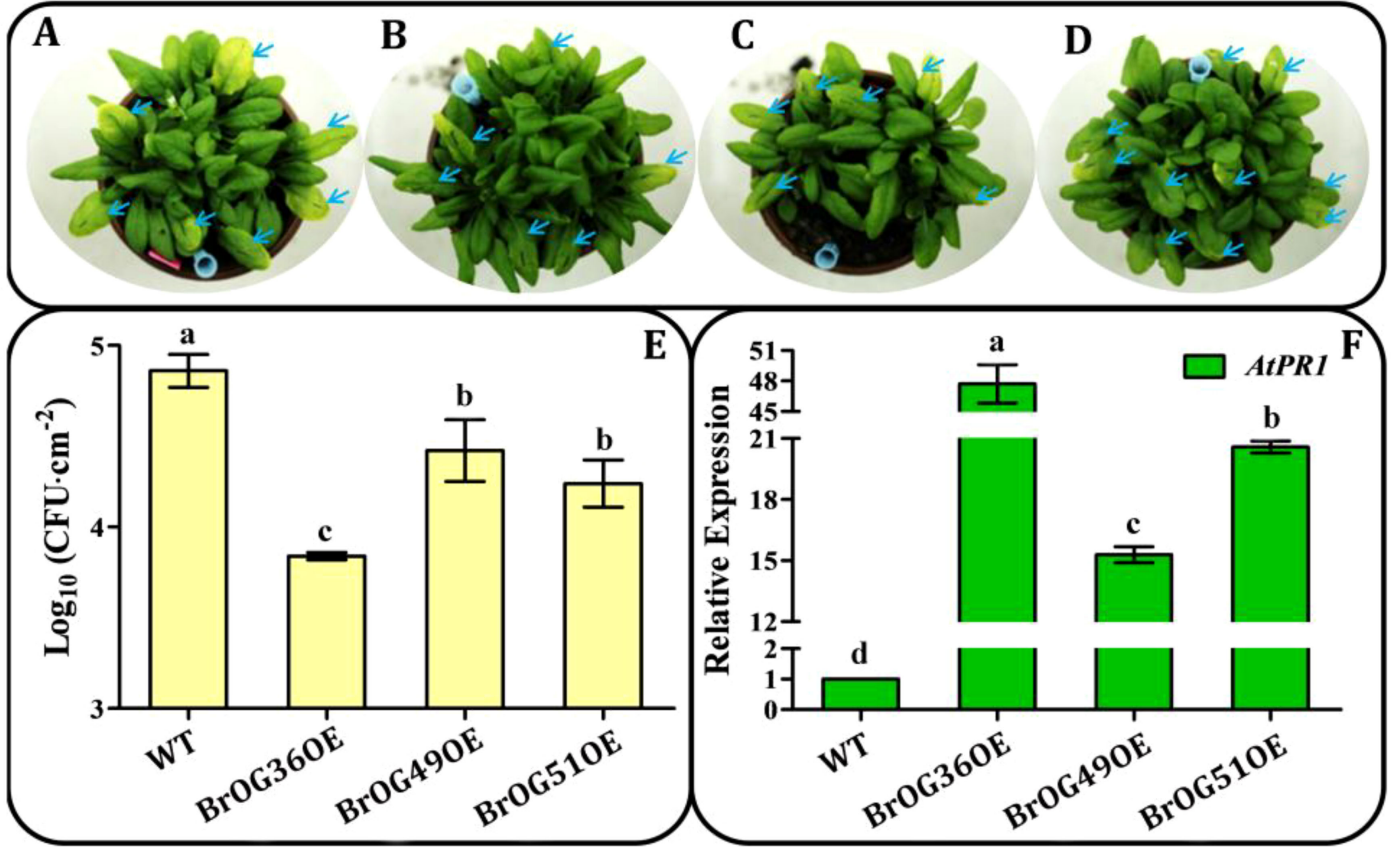
Growth of *P. syringae* pv. *tomato* (*Pst*) DC3000 on WT and BrOGsOE lines at 3 dpi. **(A-D)** WT, BrOG36OE, BrOG49OE, and BrOG51OE lines inoculated with *Pst* DC3000 (OD_600_ = 0.0002) at 3 dpi. **(E)** Bacterial growth in WT and BrOGsOE lines inoculated with *Pst* DC3000. Quantification of colony-forming units (CFUs) at 3 dpi. **(F)** *Pst* DC3000-induced *AtPR1* expression was analyzed at 3 dpi by qRT-PCR. *AtPP2AA3* gene was used as the internal reference. Different letters represent significant variances (*p* < 0.05) shown by one-way ANOVA with Duncan’s multiple-range test. All data are shown as mean ± SE of three biological replicates.

Specific *OGs* have been found to be crucial for responses to biotic and abiotic stressors (Fakhar et al., 2023). In the current overexpression library, 27.34% of the BrOGsOE lines showed no phenotypic differences relative to WT (**Figure 2**). These 35 transgenic lines were hypothesized to respond to biotic or abiotic stressors. Thus, 24 of the 35 BrOGsOE lines with no significant phenotypic differences were selected for challenge with a pathogen (*Pst* DC3000). Most BrOGsOE lines showed disease symptoms similar to those of the WT at 3 days post-inoculation (dpi) after *Pst* DC3000 infection. However, BrOG36OE, BrOG49OE, and BrOG51OE developed disease symptoms that were either milder or less severe than those of the WT (**Figures 4A–D**). This suggests that the bacterial load in BrOGsOE lines was less than that in WT plants (**Figure 4E**). The transcription levels of the salicylic acid (SA) signaling pathway marker gene *AtPR1* were compared between WT and BrOGsOE plants at the time of *Pst* DC3000 infection, as SA-mediated response is essential for protecting *Arabidopsis* against *Pst* DC3000. Significantly higher levels of *AtPR1* transcripts were observed in infected BrOGsOE plants at 3 dpi compared to the infected WT plants (**Figure 4F**), seen in lower bacterial growth and fewer disease symptoms in the BrOGsOE lines. These findings indicated that immunity could be induced in *Arabidopsis* by *BrOGs*, which in turn enhanced the durability of the innate immune system through the maintenance of defense mechanisms.

### 3.4 Screening of salt stress-response BrOGsOE lines

**Figure 5:**
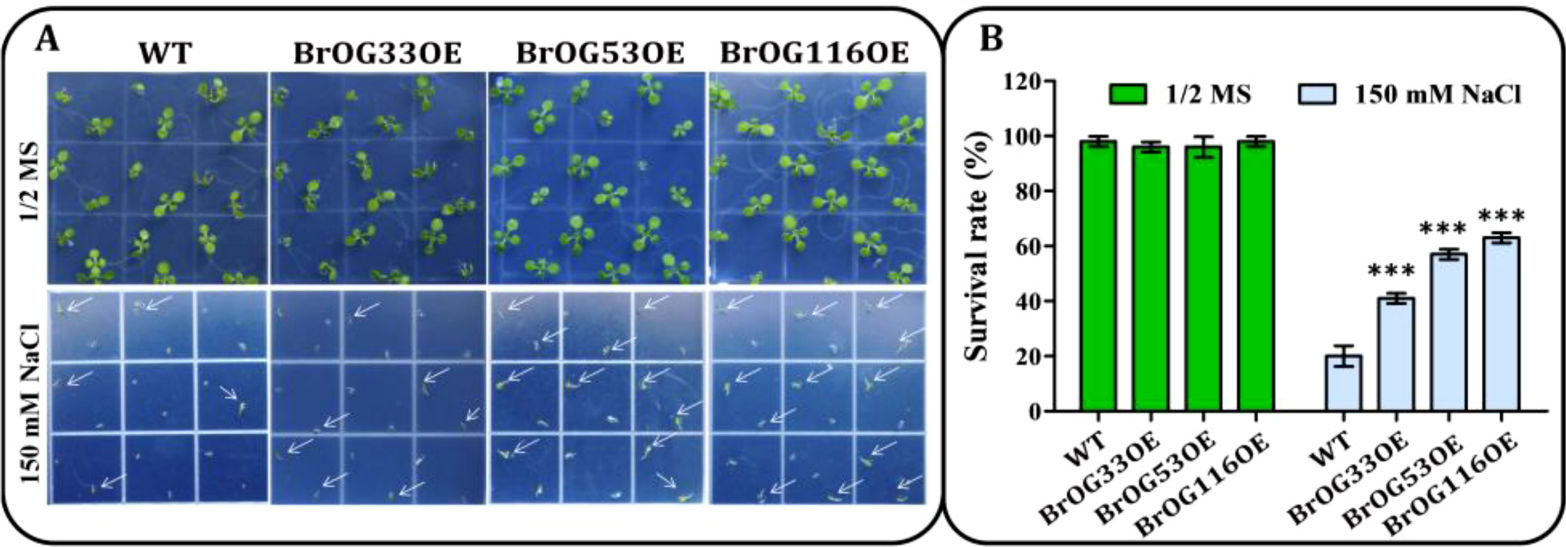
Salt stress resistant BrOGsOE lines. **(A)** Phenotypes associated with salt stress. Seeds of the WT and BrOGsOE lines were cultivated on 1/2 MS agar medium enriched with 0 or 150 mM NaCl, then placed at 4°C for 3 d Images were captured at 9 d after incubation at 22°C. **(B)** Survival rates. Asterisk (***) indicate a significant difference (*p* < 0.001) from the WT, shown by Student’s *t*-test. All data are shown as mean ± SE of three biological replicates; at least 16 seedlings were scored for each replicate/genotype.

Salt stress is an important abiotic stress that can markedly restrict the productivity of plants (Qin et al., 2017). To define the functions of *BrOGs* in response to salt stress, WT and BrOGsOE seeds were cultivated on 1/2 MS medium plates containing 0 or 150 mM NaCl. It was observed that many seedlings appeared bleached and dead after growth on 1/2 MS medium with 150 mM NaCl, in contrast to 1/2 MS medium without NaCl. The survival rate of most BrOGsOE lines was similar (12 BrOGsOE lines) or more sensitive (5 BrOGsOE lines) to that of WT seedlings (**Supplementary Figure S3**). A total of 7 BrOGsOE lines displayed enhanced tolerance to salt stress when compared to WT, such as BrOG33OE, BrOG53OE, and BrOG116OE, which possessed the highest survival rates (**Figure 5**; **Supplementary Figure S3**). The current analysis clearly showed that *BrOGs* were positively or negatively involved in the salt stress response, indicating the vital roles of *BrOGs* in plant adaptation to salt stress.

### 3.5 Screening of heat stress-response BrOGsOE lines

**Figure 6:**
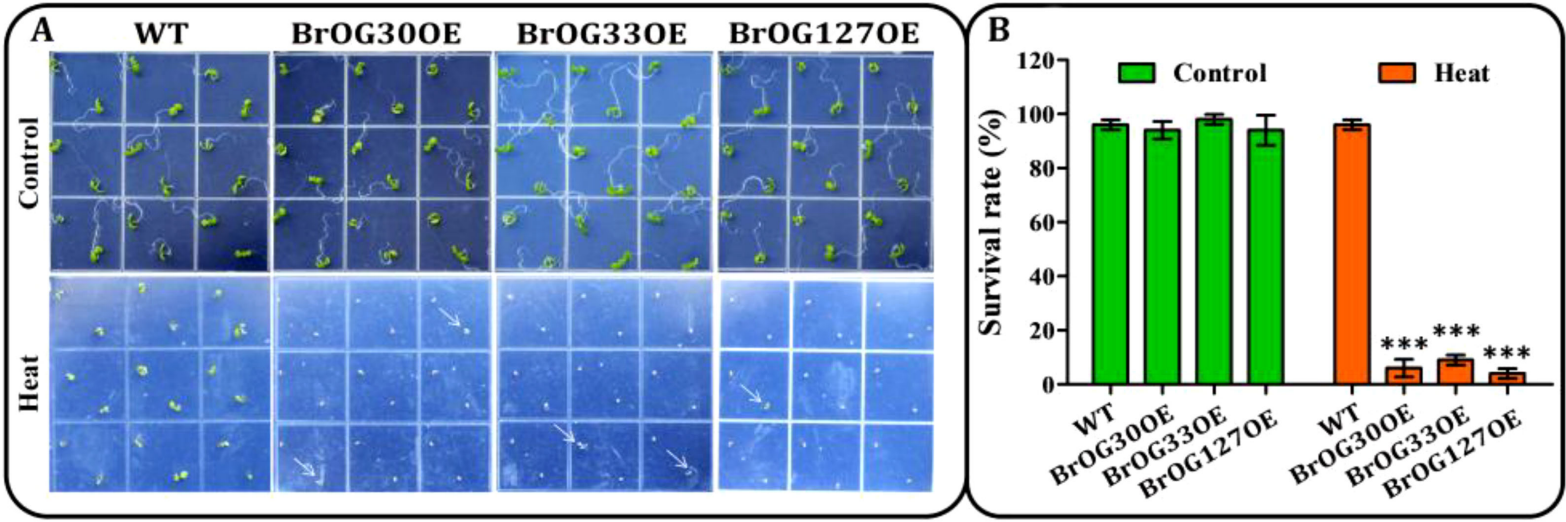
BrOGsOE lines sensitive to basal heat stress. **(A)** Heat stress phenotypes. Seedlings were incubated for 3 days at 4°C followed by 22°C for 30 h, then heated at 45°C for 2 h and recovered and allowed to grow at 22°C for 6 d before imaging. Untreated plants cultured at 22°C were depicted as controls. **(B)** Survival rates. Asterisk (***) indicate significant difference (*p* < 0.001) from the WT by Student’s *t*-test. All data are shown as mean ± SE of three biological replicates; at least 16 seedlings were scored for each replicate/genotype.

Heat stress has a significant impact on agricultural crop productivity, and is especially relevant in current conditions of climate change (Weng et al., 2014). Transgenic lines and WT seeds were cultivated on plates at 4°C for 3 days followed by 22°C for 30 h. The seeds were then heated for 2 h at 45°C and kept at 22°C for 7 days. No significant differences in the heat stress-response were observed between the 11 BrOGsOE lines and the WT (**Supplementary Figure S4**). Importantly, the survival rates of the remaining 13 BrOGsOE lines were all markedly decreased under heat stress treatment (**Figure 6**; **Supplementary Figure S4**). The BrOG30OE, BrOG33OE, and BrOG127OE lines were the most sensitive to heat stress (**Figure 6**). These findings suggested that these transgenic lines reduced their basal heat stress tolerance.

## 4 Discussion

The application of genetically modified plants, especially transgenic lines, is considered an ideal way to explore gene function. Recent developments in biotechnology have significantly reduced the generation times of these plants. The present study established an overexpression library for *Arabidopsis* using 128 *BrOGs* from Chinese cabbage. This led to the successful generation of visible transgenic lines through the overexpression of *BrOGs* in *Arabidopsis*. These genes show no sequence similarities with *Arabidopsis* genes, thus providing a solid basis for assessing the function of *BrOGs* (Jiang et al., 2018). Similarly, previous study assembled a cotton-FOX-*Arabidopsis* library that contained 6,830 transgenic lines of *Arabidopsis* resources enabling the identification of gene functions in cotton (Li et al., 2020). Moreover, approximately 6,000 transgenic *Arabidopsis* lines were generated using the iFOX-Hunting system, revealing a substantial percentage of lines with complete *B*. *napus* transgene insertions and facilitating basic insights into the high-throughput analysis of gene functions in *B*. *napus* (Ling et al., 2018). Thus, the *Arabidopsis BrOGs* overexpression library not only served as a platform for exploring the unknown functions of *BrOGs* but also provided the plant materials needed to examine the mechanism of action of such genes.

In terms of the phenotypic variation between Chinese cabbage and *A. thaliana*, this study focused primarily on two traits, namely, leaf shape and flowering time. According to the results, 72.66% of the transgenic lines in the T_2_ generation showed marked phenotypic differences (**Figure 2**). To further confirm the phenotypic variations caused by transgene expression, the *BrOGs* in BrOGsOE lines were evaluated at the DNA and expression levels. Variations in leaf morphology, including wavy leaves, serrated margins, hairy leaves, upward or downward-curved leaves, and multiple leaves, demonstrated a clear relevance between these *Arabidopsis* transgenic lines and Chinese cabbage-specific leaf traits. This suggests that *BrOGs* could be associated with the specific leaf characteristics of Chinese cabbage. Moreover, 49% of the transgenic lines showed delayed flowering, with several of the BrOGsOE lines sharing this trait with other types. A recent study demonstrated that the overexpression of *BrOG37* (*BOLTING RESISTANCE 2*, *BR2*) delayed flowering in Chinese cabbage, and further investigation showed that *BR2* positively regulates bolting resistance *via* the vernalization pathway (Zu et al., 2024). These results, therefore, support the hypothesis that *OGs* may have similar functions in different plant species. Flowering time regulation could be influenced by various signaling pathways, and the phenomenon of delayed flowering time could lead to additional phenotype variations that have been reported in many *Arabidopsis* lines (Griffiths et al., 2006; Domagalska et al., 2007). Furthermore, Chinese cabbage usually requires vernalization and photoperiodism to promote flowering and bolting (Elers and Wiebe, 1984). This study predicted that *BrOGs* associated with delayed flowering may negatively regulate flowering in Chinese cabbage, making it resistant to bolting, and thus contributing significantly to the understanding of the mechanism controlling flowering control in preventing Chinese cabbage from flowering prematurely. The results indicated that *BrOGs* are effective genetic resources for elucidating the complex genetic mechanisms underlying variations in morphological characteristics, such as leaves and flowering time, in Chinese cabbage. Moreover, such *BrOGs* may also act as critical factors in the evolution of specific traits in Chinese cabbage. The mechanisms underlying delayed or early flowering, changes in leaf shape, and increased leaf numbers warrant further research.

Orphan genes have been found to possess crucial roles in biotic interactions and environmental responses in various plants (Jiang et al., 2022). *Pst* DC3000 infects hundreds of taxonomically diverse plant species, and the potential to cause disease in *A. thaliana* rendered it a suitable model for investigating plant-pathogen interactions (Thilmony et al., 2006; Nomura et al., 2012). In this study, 3 of 24 transgenic lines were resistant to *Pst* DC3000 infection and showed higher expression of *AtPR1* and fewer bacteria compared with the WT plants. Similarly, the Brassicaceae-specific gene, *Enhancer of Vascular Wilt Resistance 1* (*EWR1*), has been shown to provide resistance against vascular wilt pathogens (Yadeta et al., 2014). Moreover, *Triticum aestivum Fusarium Resistance Orphan Gene* (*TaFROG*) provides resistance against the mycotoxigenic fungus *Fusarium graminearum* (Perochon et al., 2015). The rice tribe-specific gene *Oryza sativa defense-responsive gene 10* (*OsDR10*) acts as a negative regulator in resistance to bacterial blight disease (Xiao et al., 2009), and the *Arabidopsis OG QQS* is down-regulated in response to *Pst* DC3000 infection (Arendsee et al., 2014). These results indicated that *BrOGs* have been recruited to regulate responses to biotic stresses. Future research should explore further specificity of resistance mechanisms and functions of *BrOGs* against various pathogens, such as *Plasmodiophora brassicae*, which may provide a source to identify novel resistance-associated genes in crops.

Several factors, such as soil salinity and high temperature, negatively impact crop productivity (Weng et al., 2014; Qin et al., 2017). Among the *BrOGs* overexpression lines, 50% were insensitive to salt stress, 20.83% showed sensitive phenotypes, and 29.17% revealed increased tolerance. Similarly, a recent study reported that approximately 70% of *A. thaliana* knockout mutants corresponded to genes of unknown function associated with unaltered phenotypes under salt stress. In comparison, 6.65% showed tolerance, and 16.52% showed sensitivity to salt stress (Luhua et al., 2013). Cross-stress analysis of stress-response mutants further indicated that some *BrOGs* display specific functions under certain stress conditions. For example, the BrOG36OE lines were resistant to *Pst* DC3000 infection, while the BrOG6OE lines were tolerant to salt stress, suggesting that these *BrOGs* may be involved in specific signal transduction pathways or networks associated with specific stress responses. These findings are consistent with those of a previous study showing that genes of unknown function are species-specific and give rise to different cellular networks (Luhua et al., 2008). Several transgenic lines (BrOG33OE, BrOG53OE, and BrOG116OE) were tolerant to salt stress but sensitive to both *Pst* DC3000 infection and heat stress. Similar reports have shown that transgenic *Arabidopsis* expresses unknown functional proteins that enhance tolerance to oxidative stress without increased tolerance to osmotic, salinity, heat, or cold stresses (Luhua et al., 2008). Interestingly, the transgenic lines (BrOG49OE and BrOG51OE) that displayed tolerance to *Pst* DC3000 were also tolerant to salt stress. Furthermore, transgenic lines (BrOG25OE, BrOG30OE, and BrOG59OE) that showed sensitivity to *Pst* DC3000 infection were also observed to be sensitive to salt and heat stress, suggesting that these genes may play crucial roles in various stress responses. Previous findings showed the genes of unknown function in *Arabidopsis* could have generalized functions against stress by the activation of multiple acclimation mechanisms (Luhua et al., 2013). *BrOGs* can thus play both general and specific roles in response to pathogen invasion and environmental perturbations, and further research on *BrOGs*’ functions can provide new insights into mechanisms underlying plant responses to biotic and abiotic stressors.

## 5 Conclusions

In this study, a *BrOGs* overexpression library was constructed and comprehensively evaluated in *A. thaliana*. Significant relationships were observed between the phenotypes of these BrOGsOE lines and the specific traits of Chinese cabbage. The proportion of the delayed flowering type was much higher than that of the early flowering type, and additional phenotypes frequently accompanied delayed flowering. Various *BrOGs* have both general and specific functions against environmental and pathogenic stresses. These findings reveal the roles of *BrOGs* in the formation of species-specific traits and responses to stress, providing an important reference for the subsequent analysis of the mechanism of action of *BrOGs*.

## Data Availability

The original contributions presented in the study are included in the article/**Supplementary Material**. Further inquiries can be directed to the corresponding authors.

## Supplementary material

Supplementary Figure 1 Schematic diagram of CaMV35S::DsRed expression vector. LB indicates left border, RB indicates right border, and MCS represents multiple cloning sites.

Supplementary Figure 2 Confirmation of *BrOGs* gene expression in BrOGsOE lines. **(A)** PCR analysis of the *BrOGs* in WT and BrOGsOE plants at the DNA level. **(B)** Analysis of *BrOGs* expression by semi-quantitative RT-PCR in BrOGsOE lines. GSPs indicated *BrOGs* gene-specific primers, and *AtActin2* represented the *Arabidopsis ACTIN2* gene primers.

Supplementary Figure 3 The survival rates of WT plants and BrOGsOE lines in response to salt stress. Significant differences (****p* < 0.001; ***p* < 0.01; **p* < 0.05) relative to the WT by Student’s *t*-test. All data are shown as mean ± SE of three biological replicates; at least 16 seedlings were scored for each replicate/genotype.

Supplementary Figure 4 The survival rates of WT plants and BrOGsOE lines in response to basal heat stress. Significant differences (****p* < 0.001; ***p* < 0.01; **p* < 0.05) relative to the WT by Student’s *t*-test. All data are shown as mean ± SE of three biological replicates; at least 16 seedlings were scored for each replicate/genotype.
