# Supplementary figures and images for: Construction and evaluation of Brassica rapa orphan genes overexpression library
Source: Front Plant Sci. 2025 Jan 22;16:1532449. doi: 10.3389/fpls.2025.1532449 (PMC11794797; doi:10.3389/fpls.2025.1532449)

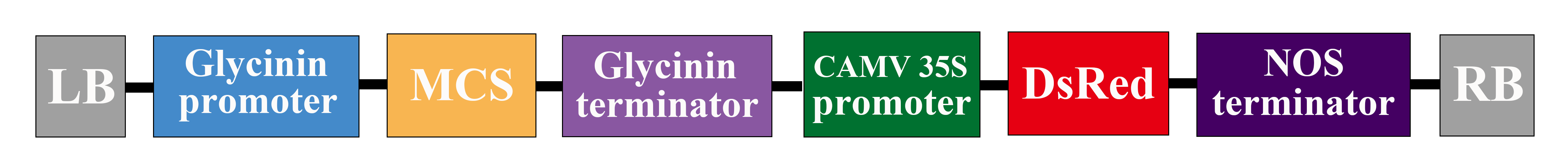

Supplement: Supplementary Figure 1 — Schematic diagram of CaMV35S::DsRed expression vector. LB indicates left border, RB indicates right border, and MCS represents multiple cloning sites. [file Image1.tif]

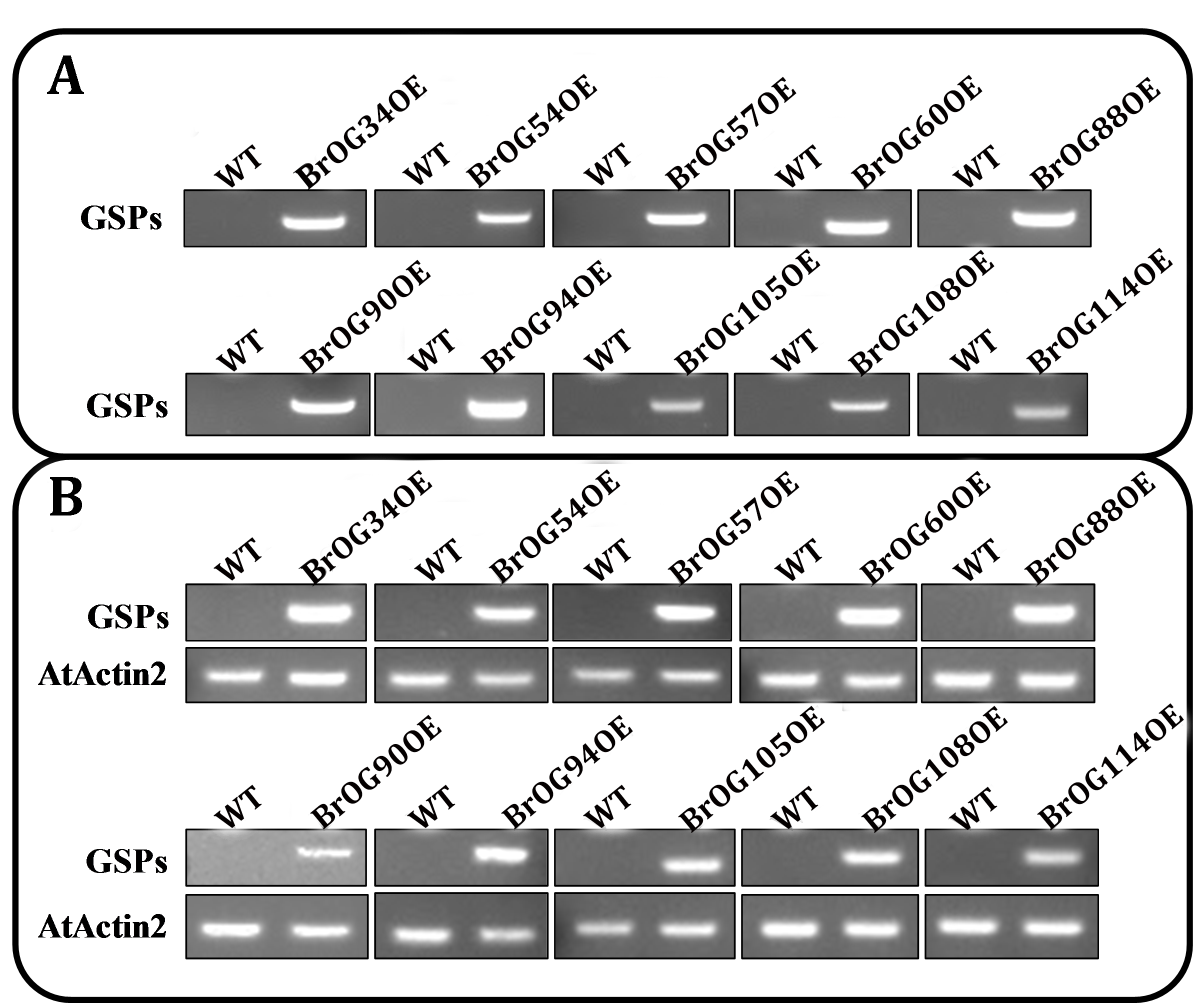

Supplement: Supplementary Figure 2 — Confirmation of BrOGs gene expression in BrOGsOE lines. (A) PCR analysis of the BrOGs in WT and BrOGsOE plants at the DNA level. (B) Analysis of BrOGs expression by semi-quantitative RT-PCR in BrOGsOE lines. GSPs indicated BrOGs gene-specific primers, and AtActin2 represented the Arabidopsis ACTIN2 gene primers. [file Image2.tif]

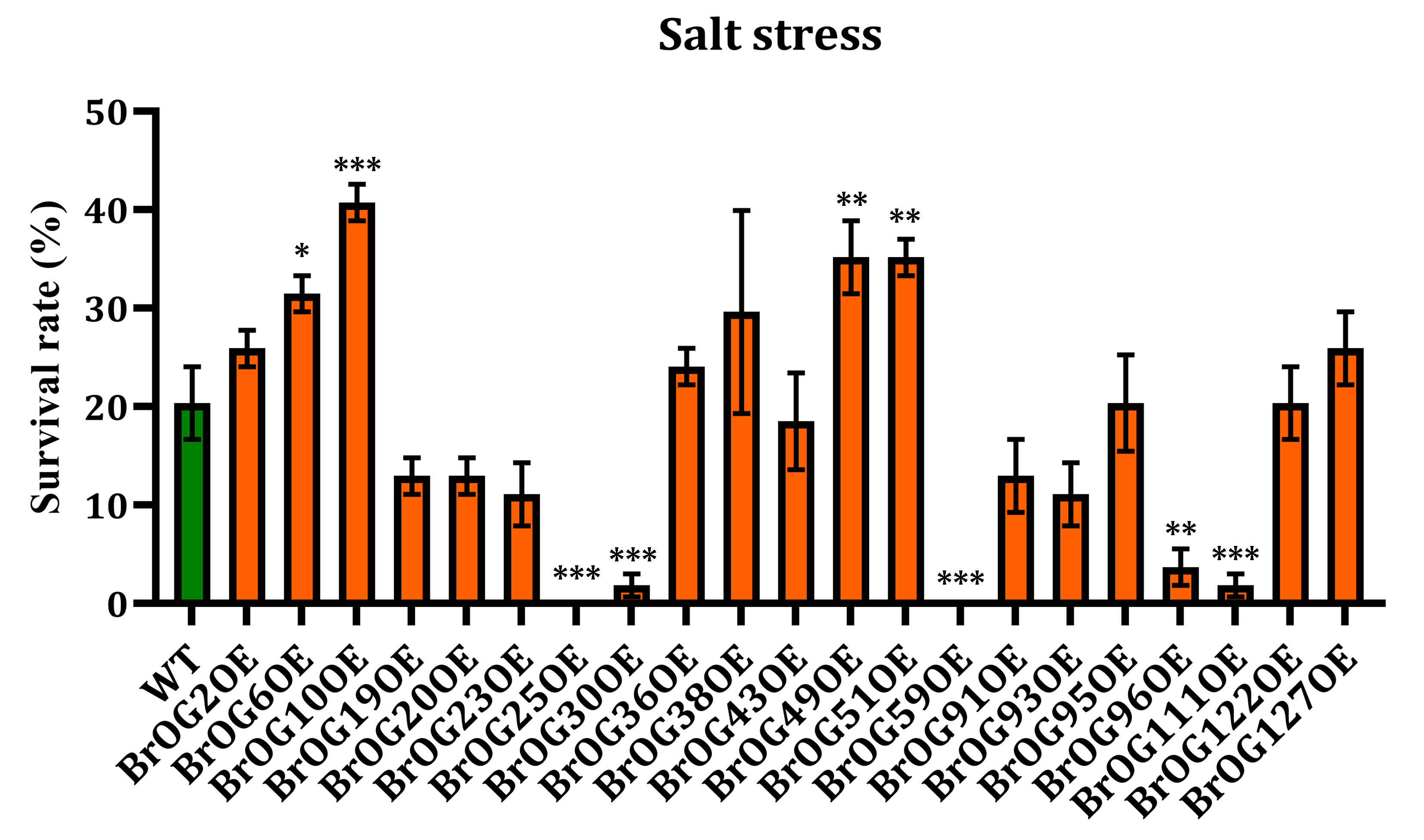

Supplement: Supplementary Figure 3 — The survival rates of WT plants and BrOGsOE lines in response to salt stress. Significant differences (***p < 0.001; **p < 0.01; *p < 0.05) relative to the WT by Student’s t-test. All data are shown as mean ± SE of three biological replicates; at least 16 seedlings were scored for each replicate/genotype. [file Image3.tif]

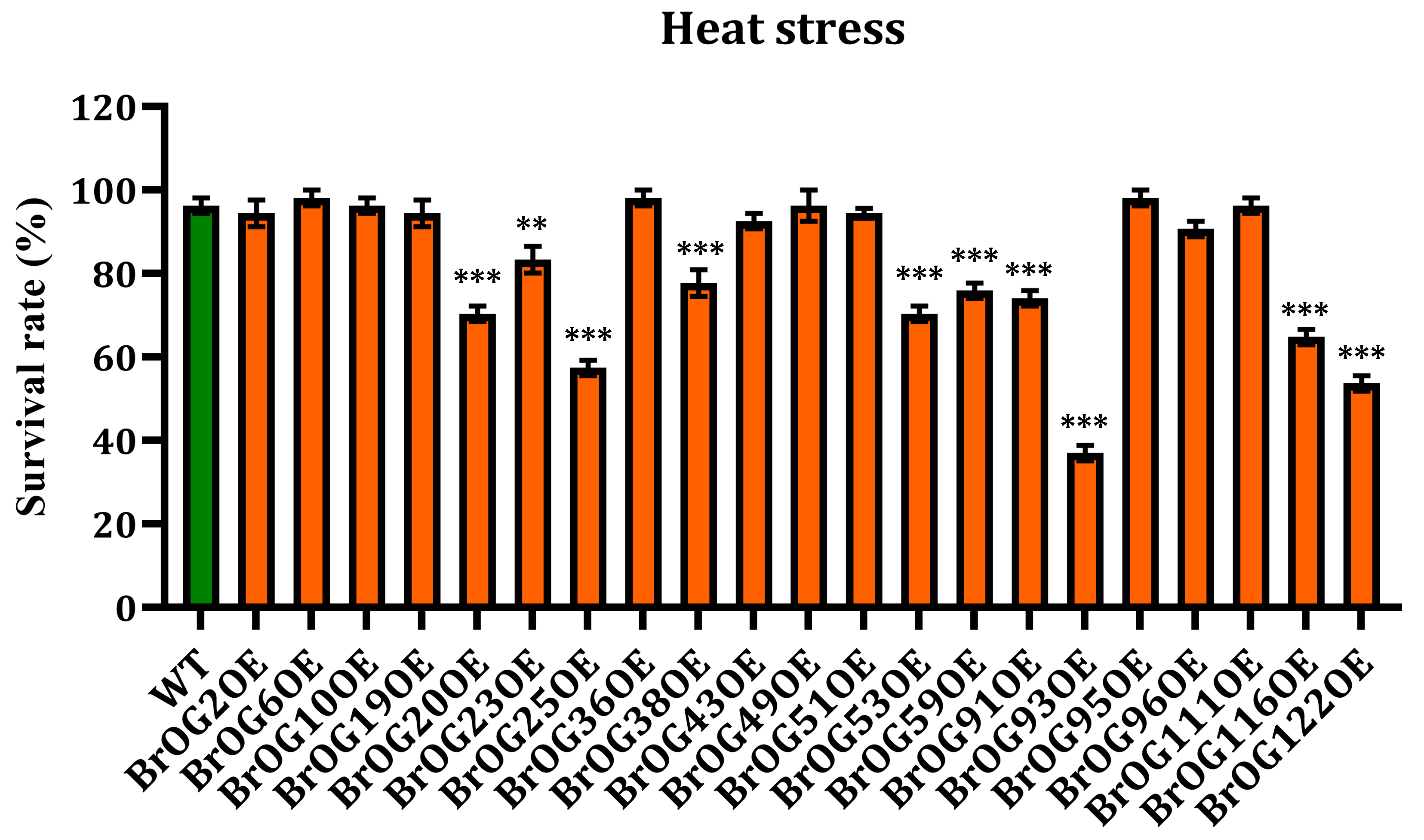

Supplement: Supplementary Figure 4 — The survival rates of WT plants and BrOGsOE lines in response to basal heat stress. Significant differences (***p < 0.001; **p < 0.01; *p < 0.05) relative to the WT by Student’s t-test. All data are shown as mean ± SE of three biological replicates; at least 16 seedlings were scored for each replicate/genotype. [file Image4.tif]
